# Loss of TRP53 (p53) accelerates tumorigenesis and changes the tumor spectrum of SJL/J mice

**DOI:** 10.18632/genesandcancer.198

**Published:** 2020

**Authors:** Jane A Branca, Benjamin E Low, Ruth L Saxl, Jennifer K Sargent, Rosalinda A Doty, Michael V Wiles, Beth L Dumont, Muneer G Hasham

**Affiliations:** ^1^ The Jackson Laboratory, Bar Harbor, Maine, USA

**Keywords:** TRP53, p53, SJL/J, tumorigenesis

## Abstract

Known as the guardian of the genome, transformation-related protein 53 (TRP53) is a well -known tumor suppressor. Here, we describe a novel TRP53 deficient mouse model on a tumor prone background—SJL/J mice. The absence of TRP53 (TRP53 nullizygosity) leads to a shift in the tumor spectrum from a non-Hodgkin’s-like disease to thymic lymphomas and testicular teratomas at a very rapid tumor onset averaging ~12 weeks of age. In haplotype studies, comparing tumor prone versus tumor resistant Trp53 null mouse strains, we found that other tumor suppressor, DNA repair and/or immune system genes modulate tumor incidence in TRP53 null strains, suggesting that even a strong tumor suppressor such as TRP53 is modulated by genetic background. Due to their rapid development of tumors, the SJL/J TRP53 null mice generated here can be used as an efficient chemotherapy or immunotherapy screening mouse model.

## INTRODUCTION

The product of the transformation-related protein (*Trp53*) is one of the most researched tumor suppressors in biomedical research, with currently more than 97,000 research/review articles published to date. Known as the guardian of the genome, the function of TRP53 protein (TP53, or, in humans, p53) has been well studied, and its functions include transcriptional regulation, DNA repair, cell cycle check-point control, apoptosis, autophagy and senescence (for review, see [1, 9]). The lack of a functional p53 gene product in humans leads to Li-Fraumeni syndrome predisposing the patient to a spectrum of early-onset cancers (for review, see [10, 12]). Importantly, it has been shown that since p53 in humans is located at a distal end of chromosome 17 (17p13.1), the loss of this piece of chromosome, and thereby p53, generally accelerates cancers [3, 13].

Since TRP53 has many crucial functions in biological systems, a number of mouse models have been made in order to elucidate TRP53 function more fully [14, 18]. Of principal note here, *Trp53* mutations have been shown to synergize with loss-of-function mutations in other tumor suppressor genes generally accelerating tumor development and progression. For example, p53 deficiency synergizes with: Rb deficiency in a conditional mouse model to produce metastatic prostate cancer [19]; NUP98 translocation in a NUP98-HOXD13–driven mouse model to accelerate complications of myelodysplastic syndrome [20]; and mutations in *Apc* to promote mammary neoplasia [21]. Hence, the disrupting of TRP53 has become a tool to accelerate the growth of tumors that develop from mutations in other tumor suppressor genes allowing more rapid and efficient study of these tumors.

Swiss Jim Lambert (SJL/J) mice, developed from three different sources of Swiss Webster mice, have become widely used owing to their high incidence of reticulum cell sarcomas. They develop lymphomas within their first year [22-31] that resemble Hodgkin’s disease [23-25] as well as B-cell non-Hodgkin’s lymphomas [26-31]. In the presence of IL-21, the tumors arising from SJL/J mice resemble human angioimmunoblastic T-cell lymphoma [22]. In addition to cancer models, these mice have been used as models for experimental autoimmune encephalomyelitis (EAE) [32], aggression [33], spontaneous myopathy in limb girdle muscular dystrophy [34], and cardiovascular disease, due to their resistance to developing atherosclerotic aortic lesions even on high fat diet [35]. The SJL/J strain is highly susceptible to mouse adenovirus 1, making it also a model for infectious disease studies [36]. Therefore, the SJL/J strain of mice is a highly valued disease model to test therapeutics for a diversity of conditions and diseases.

We previously used these mice to test to what extent the chemotherapeutic 2-deoxy-D-glucose (2DG) can alleviate the tumor burden of SJL/J mice exhibiting terminal stages of cancer [37]. 2DG is a structural analog to glucose and blocks glycolysis leading to intracellular ATP depletion, sensitizing tumor cells to radiation therapy and chemotherapy [38]. However, 2DG at high doses show hypoglycemic and adverse cardiac effects, and at tolerable doses fail to show a significant antitumor effect in many *in vivo* experiments in both mice and humans [39]. Our 2DG studies in SJL/J mice were primarily conducted to test to what extent this toxic chemotherapeutic could have reduced adverse effects when combined with other compounds. At doses that did not elicit adverse effects, 2DG alone given to mice with a SJL/J background was able to significantly shrink tumors [37]. However, the tumors develop resistance to 2DG after four weeks, after which the tumor growth re-emerges [37]. Although the penetrance of spontaneous tumorigenesis in SJL/J is >95%, the time it takes to develop such tumors is ~one year, with a range between nine months to 1.2 years [24]. This protracted pathogenesis means that it is difficult to generate sufficient cohorts of mice to be tested in a timely fashion. Therefore, to address this challenge, we hypothesized that removing *Trp53* would accelerate tumor development, and thereby enable the ability to test the efficacy of novel combinations of chemotherapies or immunotherapies within a more operationally convenient timespan.

Here, we generate a TRP53 null SJL/J mice using CRISPR Cas9 with two guide RNAs aimed at deleting exon 4 of the *Trp53* gene. Upon analysis, TRP53 was expressed in +/+ mice, reduced in heterozygous mice, and absent in -/- mice. Homozygous null mice showed a significantly shorter time of onset of tumorigenesis, and a reduced survival, with tumors being detected as early as 11 weeks of age, faster than all the other models commonly available. However, we also found that the ablation of TRP53 in SJL/J mice shifted the tumor spectrum to thymic lymphomas, testicular teratomas and rhabdomyosarcomas rather than the typical Hodgkin’s/non-Hodgkin’s lymphomas that SJL/J wild type mice develop. This unexpected shift in tumor spectrum to thymic lymphomas is observed with TRP53 mutations in other strains such as C57BL6/J, 129S1, BALB/c, FVB/NJ, and C3H/J, suggesting that deregulation of TRP53 leads to specific types of cancers depending upon the genetic background [14-18]. We further interrogated the haplotypes of these strains, comparing tumor-prone TRP53 null strains *versus* the non-tumor-prone strains, and found that a majority of the alleles that modulate the tumor incidence rate have either cancer, immune system or DNA-repair related functions. The immune system and DNA repair systems are known to be critical in regulating tumorgenesis. This result suggests that not even a strong tumor suppressor gene such as *Trp53* is canalized, and the genetic background will modify the resulting neoplasticity.

## RESULTS

### Generation of Trp53-/- SJL/J mice

Based on the ENSEMBL database (Release 98), exon 3 of TRP53 is the first exon that is sufficiently large enough to generate guides that can target the gene. Furthermore, functional domains of TRP53 are further downstream of this exon, thus targeting this exon to introduce a frame shift mutation is anticipated to lead to a null mutation. According to the ENSEMBL database, *Trp53* transcripts contain 5’UTR sequences that include ATG sequences that could be putative translational start sites. Thus, herein exon 3 is designated as exon 4, assuming that upstream starts sites yield an additional exon.

**Figure 1:**
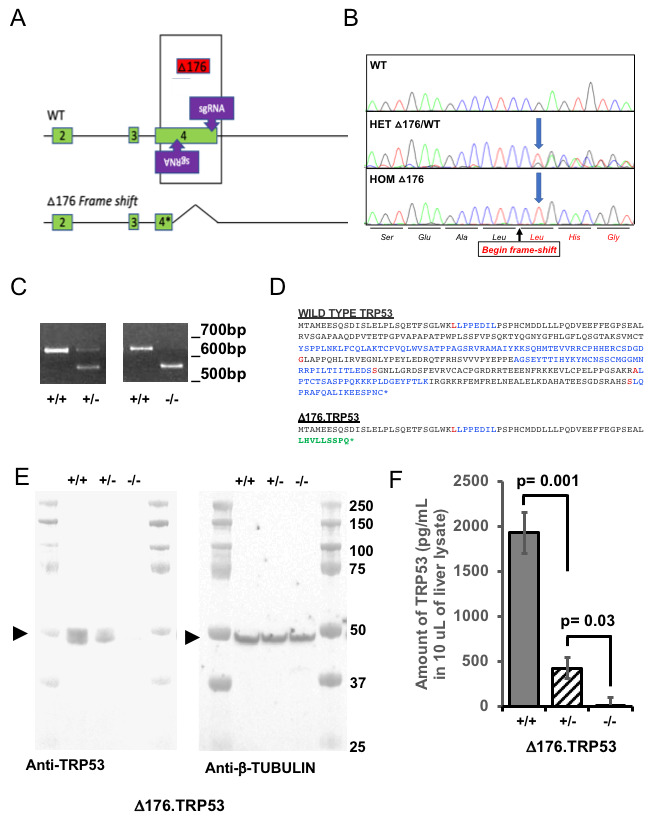
Generation of SJL/J ∆176.Trp53 mice. **A**. Design of the guides (sgRNA) targeting exon 4 that resulted in a 176 base pair deletion (∆176) (WT: wild type). **B**. Sequencing chromatograms that show the wild type (WT) littermate, the heterozygote mutant (HET∆176) and the homozygous null mutant (HOM ∆176). **C**. PCR genotyping assay that shows the wild type (+/+), heterozygous (+/-) and null (-/-) mice. **D**. The predicted amino acid sequence resulting in the deletion and generation of a stop codon. Blue and black letters show the amino acid sequence from alternating exons; red letters show predicted amino acid overlap splice site and green letters show the new amino acid sequence as a result of the deletion and subsequent frameshift and stop codon (*). **E**. Western blot of TRP53 and β-TUBULIN (loading control) showing the absence of the protein in the liver in the -/- mouse, and reduced amount of protein in the +/- mice (*n* = 3 experiments). Arrowheads indicate the position of the TRP53 and tubulin protein. **F**. Quantification of the amount of TRP53 protein by ELISA in the +/+ (gray bar), +/- (hatched bar) and -/- (white bar) mice (*n* = 4, 2 duplicate experiments and at 2 concentrations). Significant differences were determined by the student t-test.

To generate p53 knockout alleles in the SJL/J mouse strain, superovulated female mice were mated and zygotes were collected for microinjection. Of the 28 resulting offspring, 15 (54%) had modifications at one or both target sites; six had deletions of the intervening sequence between each guide and thus considered “dropouts.” Founder dropout candidates were crossed with wild type SJL/J mice and N1 heterozygotes were genotyped to characterize the mutant alleles. One allele was ultimately selected to establish a new mutant mouse strain (Figure 1A, 1B), designated SJL/J-Trp53<em2Mvw>/Mvw JAX stock 33940 (i.e. a 176bp deletion; ∆176). Genotyping and characterization of mutant alleles was accomplished using PCR (FWD: 5’- TCCAGACTTCCTCCAGAAGATA, REV:5’-CCTCTGTGCTTGGCTTCA) and Sanger sequencing. The wild type PCR product is 624bp and the ∆176 mutant allele results in a 448bp amplicon (Figure 1C).

The mutant allele was maintained in heterozygous mice. Heterozygous mice were mated to obtain the null, heterozygous or wild type alleles. Breeding outcomes showed that the allele frequency was below expected Mendelian ratios for the homozygotes at 1:3:1=wild type:heterozygous:null alleles (data from 10 litters, with litter sizes of ~ 5 mice). However, all mice (i.e., null, heterozygous, wild type) survive through weaning at a frequency similar to that of the parental SJL/J strain.

### Detection of TRP53 protein

From the nucleotide sequences, it is predicted that the ∆176 allele should yield a stop codon after 69 residues (predicted 7.8kDa), leaving the transactivation domain intact but deleting all domains downstream (Figure 1D). Intact TRP53 is predicted to be 42.5kD, however, the protein appears around 53kDa [5]. The livers, the largest and least affected organ, from a homozygous null, heterozygous and wild type littermate from a heterozygous ∆176/+ mating pair were isolated and lysates probed via western blot with a monoclonal antibody raised against serine 20 of human TRP53, which cross reacts with mouse TRP53. Western analysis showed two bands of proteins ~50kD (Figure 1E) as observed previously [5]. Our western blot observation and analysis of the ∆176 allele showed no detectable protein, suggesting that the protein might be degraded since the antibody raised against serine 20 should have been able to detect the truncated protein. The heterozygotes for this allele produce less WT TRP53 than that observed in the wild type littermates (β-tubulin was used as a loading control (Figure 1E)). In order to confirm the observed absence of the protein in the ∆176 mutant, we performed an ELISA using antibodies directed towards the N-terminus to confirm the absence of the protein in mutant mice and to quantify the amount of protein in wild-type and heterozygous mice (Figure 1F). The ELISA confirmed the western blot results, revealing that the amount of TRP53 in the livers (10uL of lysate) was 1929 pg/mL in +/+, 422 pg/mL in the +/- and 13 pg/mL in the -/-. Considering the standard error of detection in the -/- mice was 88 pg/mL, the amount in the null can be assumed to be zero.

**Figure 2:**
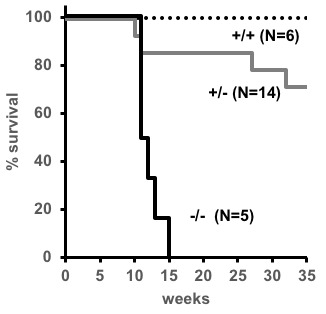
Survival of SJL/J ∆176.Trp53 mice. Graph showing a survival curve of -/- (black line), +/- (gray line) and +/+ littermate (dotted line) mice within the first 35 weeks. See text for statistical details.

### Survival of Trp53-/- and Trp53+/- mice

Mice carrying the ∆176 allele were further characterized for tumor spectrum and survival. The absence of TRP53 significantly (*P* = 0.02) shortens the lifespan of the mice from ~one year to a median of 12 weeks (Figure 2). At 35 weeks, all of the homozygous null mice (*N* = 5) and four of 14 heterozygous mice had died, but none of the wild type mice (*N* = 6) had died. Three of the 10 heterozygous mice that lived showed signs of tumorigenesis. The reduction in survival has been observed before in at least five other strains of mice with TRP53 mutations, suggesting that regardless of the genetic background, the loss of TRP53 reduces survival [14-16,18].

**Figure 3:**
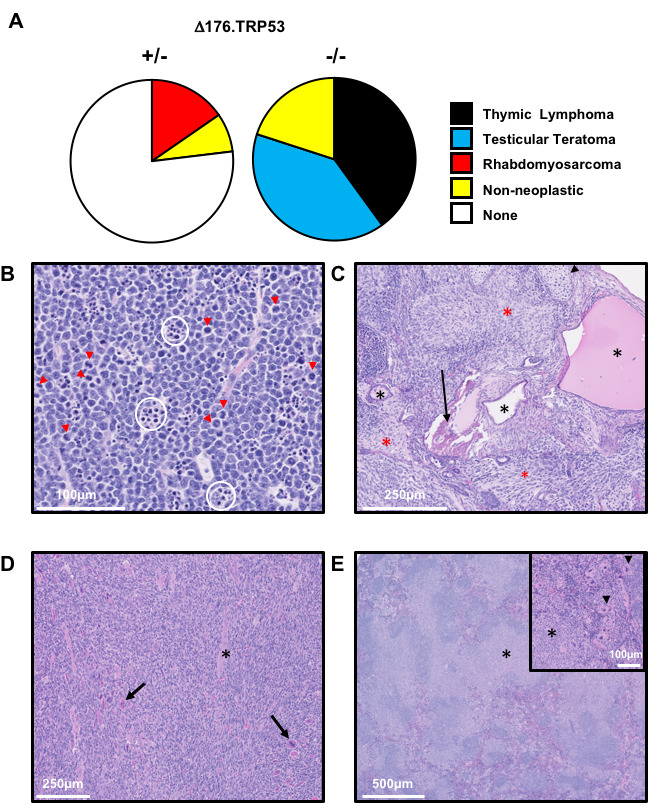
Tumor spectrum of SJL/J ∆176.Trp53 mouse. **A**. Pie charts showing the tumor spectrum distribution of the +/- and -/- mice after 35 weeks. **B**. Hematoxylin and Eosin (H&E) staining of a thymic lymphoma showing cells with high mitotic rate (red arrows) and apoptotic cells (circled in white). **C**. H&E staining of the testicular teratoma showing neoplastic tissues from ectoderm (neuropil, red asterisks), mesoderm (bone, arrow; cartilage, arrowhead), and endoderm (black asterisks). **D**. H&E staining of a rhabdomyosarcoma showing neoplastic cells that are primarily spindle cells, with a smaller proportion of round cells, as well as some multinucleated cells (arrows) and strap cells (asterisks). **E**. Splenomegaly was associated with ∆176.Trp53 -/- or +/- mice. Closer examination shows plasmacytosis (asterisks), and an increase in megakaryocytes (arrowheads) as seen by H&E staining.

The SJL/J ∆176.*Trp53*-/- mice have shown a faster development of neoplasticity than the tumor prone model BALB/c, or tumor resistant models, such as C57BL/6 (Table I) which suggests that the genetic background can modulate the phenotype even of a strong tumor suppressor such as TRP53.

### Tumor spectrum of the TRP53 null mice

No tumors were detected or observed in the wild type littermates of the mice at 35 weeks of age. TRP53 null (-/-) SJL/J mice developed thymic lymphomas (40%), testicular teratomas (40%) or were found dead without any visible tumors (20%) (Figure 3A). The heterozygous (+/-) mice developed sarcomas (15%) or were found dead without tumors, and therefore, non-cancer related cause of death (7%) (Figure 3A); none developed thymic lymphomas or testicular teratomas. There was no gender bias in the development of thymic lymphomas, and only half of male SJL/J ∆176.*Trp53*-/- mice developed testicular teratomas. A shift to thymic lymphomas is consistent with the effects of *Trp53* nullizygosity in other strains [14, 16-18],

**Figure 4:**
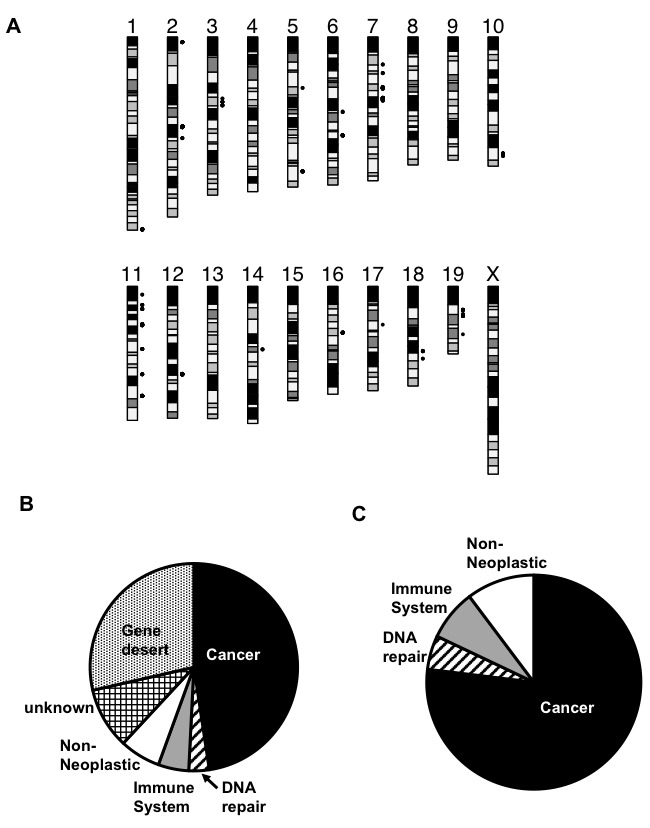
Patterns of strain haplotype sharing localize candidate regions harboring *Trp53*-/- modifiers. **A**. The standard house mouse karyotype is displayed. Gray dots denote the positions of genomic intervals where the pattern of haplotype sharing among strains mirror trends in their Trp53-/- tumor spectrum. **B**. Pie chart showing the function/pathology associated with at least one gene found in the haplotypes as observed in A. **C**. Pie chart showing the function/pathology after removing the haplotype that had either no known genes or genes with unknown function.

**Table 1:** Time of onset of neoplasias in Trp53-/- mice

Strain	Mean time of onset neoplasias in weeks	incidence rate of thymic lymphomas	Reference
C57BL/6	20.0	0.77	Donehower, et al [16]
C57BL/6J	17.2	0.79	Jacks, et al [14]
129S/J	18.0	0.65	Olive, et al; Donehower, et al [18, 17]
BALB/c	15.4	0.54	Kupperwasser, et al [15]
SJL/J	12.0	0.40	This Study

Upon closer examination of the major types of tumors derived from +/- and -/- mice, the following was observed (Figure 3B-3E): The thymic lymphomas exhibited canonical neoplastic round cells (Figure 3B) with a high mitotic rate (red arrowheads) of >20 mitotic figures per high powered field (hpf). Necrosis was minimal, but there were numerous apoptotic cells (white circles) throughout the mass. The mice also showed variable splenomegaly due to infiltration by small clusters of neoplastic lymphocytes. These features are typical of *Trp53*-/- tumors observed in previous models [14, 16].

The testicular tumor masses which were isolated from -/- mice were classical teratomas, comprised of neoplastic tissues from all three germ layers. Accordingly, the tumors were composed of a mixture of cell types (Figure 3C): ectoderm (neuropil, red asterisks), mesoderm (bone, arrow; cartilage, arrowhead; adipose tissue and muscle), and endoderm (squamous to columnar ciliated epithelium with goblet cells consistent with respiratory epithelium, black asterisks). The neoplastic cells in all of the teratomas had a high mitotic rate (>20 mitotic figures/hpf), and also showed areas of necrosis and/or hemorrhage.

The third type of tumor observed in +/- mice but not in -/- SJL/J mice were rhabdomyosarcomas (Figures 3D). These tumors were composed of neoplastic cells that were primarily spindle cells, with a smaller proportion of round cells, as well as some multinucleated cells (arrows) and strap cells (asterisks). The neoplastic cells have a high mitotic rate (>10 mitotic figures/hpf) and mild necrosis was seen in the tumor. The spleens were large due to plasmacytosis (asterisks), and the red pulp have a marked increase in megakaryocytes (arrowheads) (Figure 3E).

In summary, TRP deficiency in a SJL/J background shifts the cancer spectrum from primarily Hodgkin’s and non-Hodgkin’s lymphoma to thymic lymphomas, testicular teratoma and rhabdomyosarcomas.

### Incidence and time-of-onset of thymic lymphomas

As compared to refractive strains such as C57BL6/J or 129S, which show >65% incidence rate for thymic lymphomas, fewer SJL/J mice developed thymic lymphomas (40%), matching more closely to the tumor prone strain BALB/c (54%) (Table I). However, thymic lymphomas in the refractive strains took longer to develop than in the cancer-prone strains. For example, the average time-of-onset of thymic lymphomas exceeded 17 weeks in C57BL6/J or 129S mice, but was only 12 weeks in SJL/J mice (Table 1). Thus, tumor prone strains have a lower propensity to develop thymic lymphomas, but develop them much faster than the more refractive strains. This suggests that the genetic background may play a role in modulating the effect of Trp53 nullizygosity. It also suggests Trp53 knockout in SJL/J mice offer a more operationally convenient timespan for the study of thymic lymphomas.

### Genomic analysis of strain haplotype sharing nominates putative candidate modifiers of Trp53-/- mice

The commonly used laboratory mouse strains are descended from a small founder pool of individuals and thus only capture a limited amount of genetic diversity. Across more than 97% of the mouse genome, the genetic variation present in classical laboratory strains can be reconciled into fewer than 10 distinct haplotypes. Thus, we reasoned that putative modifiers of the strain-specific *Trp53*-/- tumor spectrum may reside in genomic regions where the refractive C57BL/6J and 129S1/SvImJ strains carry the same haplotype, but where the susceptible SJL/J and BALB/cJ strains both carry distinct haplotypes. Included in the tumor resistant phenotype group are also FVB/NJ and C3H/J, which according to JAX repository data, develop a similar tumorigenic phenotype as the C57BL6/J mice (JAX Stock # 002899 and #002547, unpublished data). Using the known haplotype patterns that are shared or distinct among strains, we identified distinct regions that could be associated with putative tumor spectrum modifiers. Overall, only 0.207% of the mouse genome (~5.45Mb) exhibited this expected haplotype profile across these six strains (Figure 4A). We grouped these haplotypes together based on the genes in which they harbored into six distinct categories, including haplotypes that harbored at least one known (i) cancer gene, (ii) DNA repair gene, (iii) immune system gene, (iv) non-neoplastic gene, (v) genes with unknown function and (vi) gene deserts (Figure 4B). After removing gene deserts and unknown function genes, we reanalyzed these data (Figure 4C). The pie charts generated show that the majority of the genes in these potential modifier regions are known to be directly associated with cancer, or function in DNA repair or the immune system, which are known to influence and modify tumorigenesis (Supplementary Figure I). Several compelling candidate genes reside in these regions, including genes *Egfr*, *Rad51b*, and *Foxp1*, which all have been shown to modify p53 tumorigenesis, as discussed below. Therefore, we have identified the likely/putative/probable genomic regions harboring *Trp53* modifiers that make strains such as BALB/cJ and SJL/J tumor prone.

## DISCUSSION

While the absence of functional TRP53 (also known as p53 in humans, TP53 in mouse) in humans causes Li Fraumeni syndrome, the loss of the distal end of chromosome 17p13.1 has been linked to a wide spectrum of early-onset cancers and acceleration of tumor growth [13]. In mice, the loss of TRP53 activity is used to generate unique mouse models for studies in transcriptional regulation, cell cycle regulation, apoptosis, and genome instability [1-3, 5-9, 13-21]. In addition, TRP53 has been mutated to rescue embryonic lethality. For example, to study non-homologous end joining in mouse models, the removal of the *Trp53* gene rescues the embryonic lethal DNA repair protein Ligase IV [43]. Of relevance to this study, TRP53 nullizygosty was successfully used to accelerate tumorigenesis in a tumor prone mouse. This is a unique model that can be used for rapid cancer and therapeutic studies. In addition, by adding this model to the library of current TRP53 mutated models, the genetic haplotypes that modify such as a strong tumor suppressor can be evaluated.

Based on this evidence, we used CRISPR-Cas9 to delete part of exon 4 (or exon 3 depending on the functional ATG site, see Figure 1A) on the TRP53 gene, with the goal that this would generate a TRP53 knock-out, since most of the domains for TRP53 function lie downstream or are a part of this exon. Such a genetic modification of this gene would lead to an acceleration of tumorigenesis and facilitate the testing of novel combinations of chemotherapeutics. As predicted, *Trp53* nullizygosity has accelerated the pace of tumor development leading to a decrease in survival. The speed with which these tumors appear is remarkably fast - 12 weeks on average, as compared to other models (Table 1). Therefore, this model is useful to study rapid tumorigenesis in mouse models.

We also showed that a decrease (null) of TRP53 changed the tumor spectrum from a non-Hogdkin’s like disease to thymic lymphoma in SJL/J mice, similar to that observed in other strains such as seen in Table I. When polymorphisms are used to classify similar mice, BALB/cJ, C57BL/6J and 129S/J are quite diverse from SJL strains [44, 45]. Yet, thymic lymphomas appear in all of these strains. The data presented here in Table I exemplify canalization, which is defined as a measure of the ability of a population to produce a similar phenotype regardless of variability of its environment or genotype. The lack of fully functional TRP53 is a powerful driver of canalization, that regardless of the genotype leads to a single major phenotype (i.e., thymic lymphomas).

However, there is a caveat to this conclusion. In the context of *Trp53* nullizygosity, the tumor prone strains SJL/J and BALB/c show the lowest incidence of thymic lymphomas but they develop much faster as compared to C57BL6/J and 129S/J, which show the highest frequency of thymic lymphomas, but they develop much slower (Table I). These results lead us to speculate that the link between Trp53 nullizygosity and tumorigenesis may not be as simple as the removal of a checkpoint in the cell cycle, ablation of apoptosis or any one simple step in a pathway, but a more complex interplay between different genomes and the propensity to develop cancer.

Based on the notion that the genome can change the phenotype of an assumed strong canalized gene such as *Trp53*, we surveyed the literature and JAX repository data, and analyzed the genomic differences based on haplotypes between tumor prone strains and tumor resistant strains. Here we have identified a number of likely/putative/probable genomic regions harboring *Trp53* modifiers that make strains such as BALB/cJ and SJL/J tumor prone. Genetic and functional tests will be required to evaluate whether and how these putative candidates may modulate the landscape of tumor formation in the absence of

*Trp53*-/-. The function of specific genes such as *Egfr*, *Rad51b*, and *Foxp1* have been shown to be modulated by *Trp53* [46-48]. A specific example is that mutations in p53 amplify EGFR family signaling to promote mammary tumorigenesis [46]. RAD51B is a homologous recombination DNA repair protein that interacts with TRP53 [49, 50], and its activity influences cell cycle checkpoint control, independent of its role in homologous recombination in breast cancer [51]. It is also known that RAD51B, being a homologous recombination protein, shares its function to maintain genome stability with TRP53 [52, 53]. FOXP1 is a transcription factor essential for the development of major organs and known to be a tumor suppressor in prostrate and breast cancers [54, 55]. In fact, *Foxp1* directly represses p53-dependent regulatory proteins in neoplastic B-cells, suggesting a strong role in immune modulation [56]. As we currently understand normal anti-cancer responses, a functional immune system is a key component that suppresses cancer [57-59]: Foxp1 controls mature B-cell survival and development, and is a regulator for CD4+ T cells [60, 61]. Thus, it is also likely that Foxp1 is an essential component that controls the lymphoid immune system, and thereby a modulator of tumorigenesis. In all of these cases, there are direct or indirect mechanisms that could be employed by these genes to modulate *Trp53*-dependent tumorigenesis.

Interestingly, known gene products of *Trp53* modifiers, such as MDM2, MDM4 and CDKN2A (p14(ARF)), did not appear in our analysis [2]. It is possible that the aforementioned genes are modifiers of *Trp53* mutations resulting in different tumor spectrums, rather than a complete loss or gain of TRP53 that changes the tumor incidence, which was the analysis done herein.

Genetically Engineered Mouse Models (GEMMS) are key to testing novel chemotherapy and immunotherapy compounds [62]. However, tumors may take more than several months to develop. To shorten tumor latency time, *Trp53* is often deleted [63, 64]. Also, the arising tumors from a TRP53 null mouse can be directly tested for drug efficacy studies (for summary see: [62]). This mouse can be used as a model to test T-cell malignancies, testicular teratomas and rhabdomyosarcomas, with the advantage that it is faster than any other available model.

The concept of whether genetic background influenced the tumor incidence or spectrum of *Trp53*-/- mice was tested decades ago: Donehower et al (1995) studied *Trp53*-/- and +/- mice and their wild-type littermates from either 129Sv or mixed C57BL/6 × 129/Sv. The 129/Sv mice showed accelerated tumorigenesis (Table I) compared with *p53*-deficient counterparts of C57BL/6 × 129/Sv genetic background, the 129/Sv mice resemble the C57BL/6 strainwith respect to tumor onset (Table I). This suggests that the genetic background indeedmodulates the TRP53 effect on tumor incidence. We have compared multiple studies (Table I), and show that different genetic backgrounds can remarkably influence tumor onset.

This study did not analyze the tumor spectrum of heterozygous mice. In addition, we reported data up to 35 weeks when a quarter of the heterozygous mice showed tumors or were found dead. Heterozygous mice express less than half the amount of TRP53 in their tissues as compared to wild type tissues. It is understood that Trp53+/- cells have the propensity to develop loss of heterozygosity (LOH) depending on the type of cell. These two aforementioned phenomena, the lowered amount of TRP53 and LOH, potentially drive the tumor onset and spectrum in the heterozygous mice [5, 6, 53]. Dissecting the two mechanisms (dose of TRP53 and LOH) that would contribute to a heterozygous cell’s propensity to become neoplastic in this tumor prone mouse model is a subject of future study.

Here, we present a mouse model of *Trp53* inactivation in the tumor prone background SJL/J. This genetic modification of *Trp53* leads to rapid tumorigenesis as compared to current models. Further, apart from the common thymic lymphomas and rhabdomyosarcomas, they also exhibit testicular teratomas. These models can now join the pantheon of *Trp53* mutations that can be used to further dissect the role of TRP53 in the context of tumor-prone models *versus* tumor-resistant models, and reveal genetic and epigenetic cancer etiologies.

## MATERIALS AND METHODS

### Mice

SJL/J mice used in this study were obtained from, and bred and housed at The Jackson Laboratory (Bar Harbor, Maine). Mice were provided food and water ad *libitum* and were housed on a 12-hour light, 12-hour dark cycle. All procedures were approved by The Jackson Laboratory Institutional Animal Care and Use Committee (IACUC).

### CRISPR mediated knock out of *Trp53*

Approximately 2-5 picoliters of Cas9 mRNA (at 100 ng/ul, Trilink), Cas9 protein (at 30 ng/ul, PNABio), and two sgRNAs (at 50 ng/ul each) were delivered by microinjection into the pronucleus of SJL/J zygotes as previously described [40]. Truncated guides (TRU-sgRNAs) [41] were designed with the assistance of online software, Breaking Cas [42], in order to minimize off-target cutting. The guides were designed to target exon 4 of the *Trp53* gene (target sites: 5’-GAGCTCCTGACACTCGGA and 5’- GCCAAGTCTGTTATGTGCA) and were made using a HiScribe kit (New England Biolabs). Microinjected zygotes were transferred to pseudopregnant females, brought to term and screened at two to three weeks of age by PCR flanking exon 4. These founder mice were backcrossed with SJL/J, and resulting mice heterozygous for the mutation were then inbred to homozygosity.

### Survival criteria

Mice were monitored daily from the time of birth and diagnosed with a tumor as soon as a visible abnormal growth appeared. The tumors were allowed to grow until the mice showed signs of illness and required euthanasia which then was recorded as the date of death. The need for euthanasia was independently verified by our Clinical and Laboratory Animal Medicine personnel. In addition, mice that were found dead were necropsied and diagnosed for the cause of death. All mice were terminated at 35 weeks of age.

### Western blotting

Snap frozen tissues were pulverized with a Spectrum Bessman Tissue Homogenizer (Fisher Scientific # 08-418-3) on dry ice. A portion of each tissue was transferred into a pre-weighed 1.5mL tube and the weight of each tissue sample was determined. The tissue samples were subsequently suspended in 100uL of chilled RIPA (Millipore-Sigma # R0278) with complete mini protease inhibitors (Roche #11836153001) and PhosSTOP phosphatase inhibitor (Roche #4906845001) per 40ug of tissue and homogenized on ice for 10 s with individual sterile pestles (Fisher Scientific #12-141-364). After 5 min on ice, homogenization was repeated. After incubating on ice for a further 30 min with a brief vortex every 5 min, the samples were spun at 10,000 × g for 10 min at 4°C. The supernatants were decanted and their protein concentration determined by the Bradford Assay (Bio-Rad # 500-0205) using BSA (Bio-Rad # 5000206) for the standard curve. Gel samples were prepared and denatured at 95°C for 5 min. 50 ug total protein per tissue was separated on 10% Bis-Tris Protein Gels (Fisher Scientific #NP0302BOX) with MOPS SDS Running Buffer (Fisher Scientific #NP0001) in duplicate. The gels were transferred to nitrocellulose (Fisher Scientific #IB3010-32) using an iBlot Gel Transfer Device (Fisher Scientific). The blots were blocked in 3% BSA in 25mM Tris·Cl, 2.7mM KCl, 137mM NaCl and 0.03% TWEEN-20 (TBST) at room temperature for 1 h. One blot was then placed in p53 (1C12) monoclonal antibody (Cell Signaling Technology #2524) diluted 1:2000 in 3% BSA-TBST and the second blot was placed in 3% BSA-TBST with a β-tubulin antibody (Millipore-Sigma #T4026) diluted 1:10,000. Both blots were agitated on a shaker at 4°C overnight. The blots were washed with TBST for 15min and then with 5 min washes then placed in 3% BSA-TBST with goat anti-Mouse IgG (H + L)-HRP conjugate (Bio-Rad # 170-6516) diluted 1:20,000 for 1 h at room temperature. After washing with TBST for 15min and then with 5 min washes, the blots were developed with SuperSignal West Pico PLUS Chemiluminescent Substrate (Fisher Scientific # PI-34087) and imaged with a G-box (Syngene).

### ELISA for *TRP53* quantification

Samples used for western blotting (see above) were quantified using the Abcam Mouse p53 ELISA Kit (Abcam # ab224878).

### Statistics and protein prediction:

For survival curves, *P*-values were calculated by log-rank (Mantel-Cox) test using Prism7 v7.0d software. Protein sizes were predicted from the Protein Calculator (v3.4) (http://protcalc.sourceforge.net/)

### Genome queries

Genome-wide haplotype strain distribution patterns were downloaded from the Mouse Phylogeny Viewer (http://msub.csbio.unc.edu/). A custom Perl script was used to identify genomic regions harboring a single shared haplotype between C57BL/6J, FVB/NJ, C3H/HeJ and 129S1/SvImJ, a second distinct haplotype in SJL/J, and a third haplotype in BALB/cJ. These genomic regions were then intersected with genes in the mm10 RefSeq database. We performed a manual inspection of the resulting gene list to nominate putative modifiers of Trp53 that could underlie observed strain differences in the tumor spectrum.

## SUPPLEMENTARY MATERIAL


