# Supplementary material for: Loss of TRP53 (p53) accelerates tumorigenesis and changes the tumor spectrum of SJL/J mice
Source: Genes Cancer. 2020;11(1-2):83–94. doi: 10.18632/genesandcancer.198 (PMC7289902; doi:10.18632/genesandcancer.198)
Supplement: Supplementary file 1 [file ganc-11-83-s001.pdf]

Loss of TRP53 (p53) accelerates tumorigenesis and changes the tumor spectrum of SJL/J mice – Branca et al

Supplementary Table1:

Supplementary Table I: Genes associated with the Haplotypes of the tumor prone versus tumor resistant mice

chr	start	stop	B6	FVB	C3H	129S1	SJL	BALBc	RefSeq	Role	Selected Literature
1	194593441	194943440	0	0	0	0	7	3	Gm39748;LOC108167784;Mir3962;Plxna2;Cd34	Cancer	https://www.ncbi.nlm.nih.gov/pubmed/25519836
2	90310110	90650109	7	7	7	7	1	6	Olfr1274-ps;Gm13769;Gm39869;Gm39871;Ptprj;LOC108168775;4933423P22Rik;Gm39870	Cancer	https://www.ncbi.nlm.nih.gov/pmc/articles/PMC5955124/
2	90720110	90900109	7	7	7	7	1	4	Nup160;Ndufs3;Gm32514;Gm39872;C1qtnf4;Agbl2;Fnbp4;Mtch2	Cancer	https://www.ncbi.nlm.nih.gov/pmc/articles/PMC4069086/
2	5080110	5250109	1	1	1	1	3	2	LOC108168737;Gm39755;Ccdc3	Cancer	https://www.ncbi.nlm.nih.gov/pubmed/31081073
2	91020110	91360109	7	7	7	7	5	4	Gm39874;Psmc3;Madd;Spi1;1110051M20Rik;Gm39875;Acp2;LOC102632821;Gm31054;Rapsn;Arfgap2;Nr1h3;A330069E16Rik;Mybpc3;Slc39a13;Ddb2;Pacsin3	Cancer	https://www.ncbi.nlm.nih.gov/pubmed/28219737
2	102350110	102380109	4	4	4	4	6	5	Trim44	Cancer	https://www.ncbi.nlm.nih.gov/pmc/articles/PMC6437891/
3	69012787	69032786	3	3	3	3	1	0	Smc4	Cancer	https://www.ncbi.nlm.nih.gov/pmc/articles/PMC5043270/
3	69172787	69222786	3	3	3	3	1	4	Arl14	Cancer	
3	65612787	65672786	5	5	5	5	6	3	Lekr1;Mir8120	Cancer	https://www.ncbi.nlm.nih.gov/pubmed/27378695
5	135970235	136130234	1	1	1	1	3	2	Zp3;Mir7035;Gm40347;Upk3bl;Rasa4;Upk3b;Lrwd1;Ssc4d;Polr2j;Dtx2	Cancer	https://www.ncbi.nlm.nih.gov/pubmed/25147919
5	51480235	51500234	2	2	2	2	4	5	Ppargc1a	Cancer	https://www.ncbi.nlm.nih.gov/pubmed/28803067
7	50636246	50686245	7	7	7	7	0	4	Nell1	Cancer	https://www.ncbi.nlm.nih.gov/pubmed/25726761
7	51516246	51586245	7	7	7	7	5	2	Gm33190;Ano5	Cancer	https://www.ncbi.nlm.nih.gov/pubmed/28902351
7	51876246	51936245	3	3	3	3	5	7	Gas2	Cancer	https://www.ncbi.nlm.nih.gov/pubmed/26358320
7	61826246	61906245	2	2	2	2	6	7	Mir344;A230057D06Rik;Mir344c	Cancer	https://www.ncbi.nlm.nih.gov/pubmed/24333578
10	119729197	119799196	7	7	7	7	0	3	Grip1os2;Grip1;LOC108167790	Cancer	https://www.ncbi.nlm.nih.gov/pubmed/28878246
11	16815934	16865933	1	1	1	1	4	2	Egfr	Cancer	https://www.ncbi.nlm.nih.gov/pubmed/16377102
11	35405934	35475933	0	0	0	0	4	2	Slit3	Cancer	https://www.ncbi.nlm.nih.gov/pubmed/27082735
11	99745934	100025933	2	2	2	2	4	6	Krtap family	Cancer	https://www.ncbi.nlm.nih.gov/pubmed/27375028
11	20555934	20635933	6	6	6	6	5	2	Sertad2	Cancer	https://www.ncbi.nlm.nih.gov/pubmed/27827314
11	56985934	57075933	4	4	4	4	5	0	Gria1	Cancer	https://www.ncbi.nlm.nih.gov/pubmed/28979808
11	99515934	99545933	2	2	2	2	5	6	Krt40;Krt39	Cancer	https://www.ncbi.nlm.nih.gov/pmc/articles/PMC6435686/
11	34265934	34315933	3	3	3	3	6	2	Dock2;4930403D09Rik;Fam196b	Cancer	https://www.ncbi.nlm.nih.gov/pubmed/26658103
14	57384187	57424186	0	0	0	0	1	2	Cryl1;Ift88	Cancer	https://www.ncbi.nlm.nih.gov/pubmed/28489570
16	42188121	42288120	6	6	6	6	2	0	Gap43	Cancer	https://www.ncbi.nlm.nih.gov/pubmed/30419922
16	41978121	42038120	6	6	6	6	3	0	Lsamp	Cancer	https://www.ncbi.nlm.nih.gov/pubmed/26844274
16	42158121	42188120	6	6	6	6	3	0	Lsamp	Cancer	https://www.ncbi.nlm.nih.gov/pubmed/26844274
17	34857454	34867453	2	2	2	2	0	4	Mir6972;Cfb;C2	Cancer	https://www.ncbi.nlm.nih.gov/pubmed/28322200
18	59042495	59102494	0	0	0	0	6	7	A730017C20Rik;Adamts19	Cancer	https://www.ncbi.nlm.nih.gov/pubmed/26634009
19	21275547	21435546	6	6	6	6	5	4	Zfand5;Gda	Cancer	https://www.ncbi.nlm.nih.gov/pubmed/30953370
7	27746246	27776245	2	2	2	2	7	5	Gm10046;Zfp60	Cartilage Differentiation	https://www.ncbi.nlm.nih.gov/pubmed/12469909
10	119819197	120139196	7	7	7	7	0	1	Helb;Grip1;Grip1os1	DNA Repair	https://www.ncbi.nlm.nih.gov/pubmed/26774285
12	79651693	79991692	6	6	6	6	0	1	Rad51b	DNA repair, Cancer	https://www.ncbi.nlm.nih.gov/pmc/articles/PMC4554751/
11	80055934	80135933	1	1	1	1	3	2	Atad5;Crlf3	Immunity	https://www.ncbi.nlm.nih.gov/pubmed/25404367
19	25175547	25195546	3	3	3	3	5	2	Dock8	Immunity	https://www.ncbi.nlm.nih.gov/pubmed/28366940
6	99491551	99701550	3	3	3	3	7	1	Gpr27;LOC108169204;Foxp1;Eif4e3	Immunity, Cancer	https://www.ncbi.nlm.nih.gov/pubmed/26654944
7	63896246	63956245	2	2	2	2	6	5	E030018B13Rik;Gm27252;LOC108167433;Klf13	Microtubule Dynamics	https://www.ncbi.nlm.nih.gov/pubmed/26948876
7	63506246	63526245	2	2	2	2	6	1	Otud7a	Neural Development	https://www.ncbi.nlm.nih.gov/pubmed/29395074
12	80151693	80311692	6	6	6	6	0	1	Gm29952;Actn1	Thrombocytopenia	https://www.ncbi.nlm.nih.gov/pubmed/25361813
7	61266246	61276245	2	2	2	2	0	4	Gm31445	Unknown	
7	36676246	36686245	4	4	4	4	5	2	Gm36722	Unknown	
7	61526246	61666245	2	2	2	2	6	4	B230209E15Rik	Unknown	
7	63996246	64016245	2	2	2	2	6	5	Gm32633	Unknown	
10	117849197	117909196	0	0	0	0	3	5	Gm32605;Gm40773;Gm32552	Unknown	
19	43645547	43655546	0	0	0	0	4	5	Gm34922	Unknown	
2	102470110	102480109	4	4	4	4	6	5			
3	62252787	62272786	4	4	4	4	7	3			
6	76011551	76031550	0	0	0	0	3	4			
6	75311551	75321550	0	0	0	0	7	3			
7	36396246	36426245	4	4	4	4	3	0			
7	51066246	51096245	7	7	7	7	3	0			
7	51096246	51166245	7	7	7	7	5	0			
7	51246246	51356245	7	7	7	7	5	0			
7	52376246	52416245	3	3	3	3	5	4			
7	62866246	63006245	2	2	2	2	6	1			
7	64476246	64496245	3	3	3	3	7	5			
10	117569197	117579196	0	0	0	0	3	5			
10	118349197	118399196	0	0	0	0	3	1			
11	7475934	7495933	2	2	2	2	3	7			
11	35015934	35115933	2	2	2	2	4	3			
18	58702495	58722494	0	0	0	0	1	4			
18	65832495	65852494	3	3	3	3	7	6			
19	26975547	27055546	3	3	3	3	5	4			
